# Resilient performance indicator virus for membrane filtration considering surface charge, hydrophobicity, and morphology

**DOI:** 10.1128/aem.00035-26

**Published:** 2026-05-11

**Authors:** Midori Yasui, Tatsuya Sakai, Takashi Hashimoto, Sadahiko Itoh

**Affiliations:** 1 Department of Environmental Engineering, Kyoto University 12918, Kyoto, Japan; 2 Research Center for Water Environment Technology, The University of Tokyo 13143, Tokyo, Japan; University of Minnesota Twin Cities, St. Paul, Minnesota, USA

**Keywords:** performance indicator virus, morphology, hydrophobicity, surface charge, surface property, membrane filtration, water treatment, virus removal

## Abstract

Highly abundant viruses in environmental waters/wastewaters—for example, Pepper Mild Mottle Virus (PMMoV), Aichi virus (AiV), and F-specific RNA coliphages—have been monitored as performance indicator viruses to evaluate the virus removal capacity of full-scale drinking and wastewater treatment systems. These viruses have also been employed in bench-scale challenge tests as performance indicators in place of pathogenic viruses. However, due to the difficulty of directly evaluating pathogenic virus behavior, the selection of appropriate indicators to ensure the safety of treatment processes remains under debate. Our study aims to identify a more resilient performance indicator virus for membrane filtration processes by examining the influence of surface charge, hydrophobicity, and morphology on removal efficiency. We measured the zeta potentials and contact angles of bacteriophage MS2, PMMoV, and AiV, and evaluated their adsorption to membrane surfaces, and removal efficiency by membrane filtration. PMMoV has a rod-shaped structure, whereas the others have a sphere-shaped structure. Although these viruses showed similar zeta potentials, their contact angles varied, indicating hydrophobicity strength in the order of MS2 < PMMoV < AiV. In filtration experiments, PMMoV showed the highest removal efficiency despite its intermediate hydrophobicity among the three viruses. AiV, the most hydrophobic virus, showed removal performance similar to that of MS2, the least hydrophobic virus. Our results indicate that the rod-shaped structure of PMMoV enhanced its removal by membrane filtration, and that the spherical viruses, such as MS2 and AiV, may serve as more resilient performance indicators.

**IMPORTANCE:**

This study combines physicochemical evaluations with discussions to propose a resilient performance indicator virus for membrane filtration processes. The charge characteristics and hydrophobicity of MS2, PMMoV, and AiV were experimentally evaluated. Although all three viruses exhibited similar surface charges, their hydrophobicity differed. However, these differences did not significantly influence virus removal by membranes. Instead, our findings highlight the critical role of viral morphology in governing removal behavior during membrane filtration. These results provide new insights into the selection of appropriate performance indicator viruses for both bench-scale and full-scale virus removal evaluations.

## INTRODUCTION

Proper management of pathogenic microorganisms is essential for ensuring the safety of water supply systems. Particularly, pathogenic viruses are challenging contaminants due to their high persistence during water treatment processes and their high infectivity to humans. The WHO Guidelines for Drinking-water Quality provide example calculations for tolerable pathogen burdens in drinking water, and that for rotavirus is 1.1 × 10^−5^ infectious virions/L (1). Consequently, daily monitoring of virus concentrations in finished water is practically infeasible because more than 100,000 L of water would have to be investigated to ensure this safety level. Therefore, to ensure the viral safety of treated water, it is necessary to establish performance targets, specifically the required total log reduction of viruses, which are the sum of the credited log reductions from individual processes within a treatment train (2–4). For viruses, although higher log reductions are typically allocated to disinfection processes, physical treatment processes, especially membrane filtration processes, have no or low credited log reductions, as their virus removal performance, estimated from surrogate water quality parameters (e.g., conductivity, TOC), tends to be low (2). However, virus concentration monitoring at pilot-scale or practical plants has revealed that membrane filtration can achieve higher levels of virus removal than those credited (5, 6).

Performance indicator viruses instead of pathogenic viruses have been applied to assess the virus removal in treatment processes, particularly physical removal treatments such as membrane filtration. In bench-scale evaluations, bacteriophage MS2 is the most commonly used indicator virus and is mentioned in the regulatory guidelines (2, 7); it is intentionally added to test waters to determine virus removal efficiencies. For pilot- or full-scale system evaluation, naturally occurring viruses have attracted attention to investigate virus removal efficiency without artificially spiking feed water. F-specific RNA coliphages, including wild-type MS2, have been employed for monitoring virus reduction at wastewater treatment facilities (8, 9). In addition, for drinking water treatment and potable reuse facilities, several highly abundant viruses have been proposed as monitoring indicators to evaluate the virus removal performance of treatment trains, including Aichi virus (AiV) (5, 10), plant viruses such as pepper mild mottle virus (PMMoV) (5, 6, 10–12), and crAssphage (5, 13–15). In full-scale systems, ideal performance indicator viruses should be highly abundant in the target water sources and exhibit removal behaviors similar to those of pathogenic viruses. However, the selection of the most suitable performance indicator virus remains under debate due to the difficulty of direct comparisons between removal values of indicator viruses and pathogenic viruses. Under such situations, selecting the least removed candidate as a resilient performance indicator is preferable to avoid overestimating pathogenic virus removal. By evaluating physicochemical factors that influence virus removal behavior, it may be possible to identify a resilient performance indicator virus that is highly resistant to removal during membrane filtration.

Surface interactions, including electrostatic and hydrophobic-driven interactions, play an important role in virus removal by physical removal processes (16). In the case of microfiltration (MF), its pore size is generally larger than viruses, meaning size exclusion cannot be relied on. Nonetheless, a certain level of virus removal ranging from 0.9 to 4.6 log reduction by MF has been reported (17–19). As summarized by Michen and Graule (20), most viruses showed negative charges under neutral pH conditions; therefore, negatively charged MF membranes showed none or less virus removal compared to positively charged membranes (17, 18). Interestingly, hydrophobic MF membranes also removed viruses even when their charges were negative (17–19). Yasui et al. (18) explained the mechanism of virus removal by hydrophobic membranes using extended DLVO (XDLVO) models, which accounts for van der Waals, electrostatic, and Lewis acid-base (hydrophilic and hydrophobic) interactions to calculate total energy as a function of separation distance. The XDLVO models revealed that hydrophobic attraction can drive virus removal when electrostatic repulsion is sufficiently weakened to allow virus particles to approach close to the membrane surface (e.g., under high ionic strength conditions) (18). Conversely, Gentile et al. (21) reported that electrostatic repulsion can enhance the virus removal during ultrafiltration (UF), where pore sizes are smaller than viruses, by generating strong repulsive forces. To discuss the effect of surface interactions between membranes and indicator virus candidates, their surface properties have to be characterized.

The surface properties of candidate performance indicator viruses remain poorly characterized. Among the candidates mentioned above, the surface properties of bacteriophage MS2, a laboratory strain of F-specific RNA coliphages, have been extensively studied. To the best of our knowledge, most studies report low isoelectric points (pI 2.2–3.9), indicating that MS2 has a negative charge under neutral pH conditions (20). In terms of hydrophobicity, which has been evaluated on a relative basis, MS2 is generally considered to be more hydrophilic than other bacteriophages, such as bacteriophage Qβ and GA (22, 23). In contrast, information on other candidates’ surface properties remains limited. Notably, it has been reported that measured surface properties can vary depending on purification methods and measurement techniques. Therefore, measurements using standardized methods are necessary to accurately interpret virus behavior.

Furthermore, the influence of virus morphology on removal performance needs to be considered. For instance, F-specific RNA coliphages, including MS2, have an icosahedral structure with a diameter of approximately 26 nm (24), which is comparable to that of some enteric viruses such as norovirus and enterovirus. However, their structure differs from that of several proposed indicator virus candidates: PMMoV has a rod-shaped structure, and crAssphage has a short-tailed structure, whereas MS2 has an icosahedral shape. These structural differences contrast with those of enteric viruses such as norovirus, rotavirus, and enterovirus, which are generally spherical. Such morphological variations may contribute to differences in virus removal behavior during membrane filtration.

Our study aims to propose a resilient performance indicator virus for membrane filtration, defined as a virus that is less readily removed. In particular, we focus on identifying key physicochemical characteristics governing virus removal, considering both surface properties and morphology. As candidate performance indicator viruses, we selected MS2, AiV, and PMMoV, which are culturable and possess different morphologies (sphere-like vs. rod-shaped). To achieve this objective, we (i) measured the surface properties (zeta potentials and contact angles) of three viruses and (ii) evaluated the effects of these surface properties and viral morphologies on virus adsorption to and removal by membranes.

## MATERIALS AND METHODS

### Virus and membrane information

We targeted bacteriophage MS2, PMMoV, and AiV as surrogates in full-scale treatments (Table 1), and used four types of membranes (Table 2). The HL-0.1 (0.1 μm pore, hydrophilic membrane, VVLP09050, Merck Millipore), HL-0.45 (0.45 μm pore, hydrophilic membrane, HVLP09050, Merck Millipore), and HB-0.45 (0.45 μm pore, hydrophobic membrane, HVHP09050, Merck Millipore) are MF membranes, and the UF-1k (Molecular Weight Cut-Off (MWCO) 1k Da, UF membrane, 14609-76-D, Sartorius) is a UF membrane. All membranes were cut to a diameter of 35 mm for adsorption testing and 71 mm for filtration testing.

**TABLE 1:** Virus information

Virus	Virus type	Genome	Shape	Diameter (nm)
MS2	Bacteriophage	ssRNA	Icosahedron	26
PMMoV	Plant virus	ssRNA	Rod shape	Short: 18 Long: 300
AiV	Enteric virus	ssRNA	Icosahedron	30

**TABLE 2:** Membrane information

Membrane	Membrane type	Pore size or MWCO	Material
HL-0.1	Flat sheet MF	0.10 μm	PVDF
HL-0.45	Flat sheet MF	0.45 μm	PVDF
HB-0.45	Flat sheet MF	0.45 μm	Hydrophobic PVDF
UF-1k	Flat sheet UF	1 kDa	PES

### Virus propagation and purification

Bacteriophage MS2 (NBRC102619, National Institute of Technology and Evaluation) was propagated with *E. coli* K12 F + A/λ (NBRC3301) in LB broth overnight at 37°C with shaking. PMMoV (MAFF104099, National Agriculture and Food and Research Organization) was inoculated into Nicotiana benthamiana and propagated. AiV (provided by Prof. Hiroyuki Katayama, the University of Tokyo) was propagated with Vero cells (JCRB9013, National Institute of Biomedical Innovation, Health and Nutrition) in 5% CO_2_ at 37 °C. Details of PMMoV and AiV propagation are described in the supplemental material. Propagated viruses were concentrated with Amicon Ultra filter devices (15 mL, 100 kDa molecular weight cutoff, Merck Millipore Ltd.) when their concentration was lower than 10^11^ virions/mL.

After concentration, viruses were purified by density gradient centrifugation. MS2 and PMMoV were purified by cesium chloride (CsCl, Wako, Japan) density gradient centrifugation as described by Torii et al. (25). Briefly, 0.5 mL of virus stock was layered on 4.25 mL of CsCl solutions, the concentration of which was determined based on the target virus buoyant density (26). Then, the tubes were ultracentrifuged (approx. 150,000 g) for 18 h at 15 °C. For AiV, two-step purification was adopted. The AiV stock was purified by sucrose density gradient centrifugation before the CsCl density separation to remove residual host debris and achieve sufficient purity of samples. The 0.5 mL of AiV stock was layered on the 4.25 mL of a 30%–60% w/v sucrose gradient layer and then ultracentrifuged (approx. 110,000 g) for 1 h at 15 °C. These purification processes were selected based on particle size distribution results; details are described in section S2.1 in the supplemental mataerial. Collected virus bands were dialyzed using Amicon Ultra filter devices (0.5 mL, 100 kDa molecular weight cutoff, Merck Millipore Ltd.) against 10 mM NaCl solution. A significant loss in virus amount was not observed based on infectious MS2 concentrations before and after the purification process (data not shown). The purity of each virus stock and the protocol for quantifying infectious MS2 are provided in the supplemental material.

### Virus and membrane zeta potential measurements

Purified viruses were dispersed in NaCl solutions of various pH values from 2 to 8 at high concentrations (10^11^–10^13^ copies/mL). The pH of solutions was adjusted with 0.1 M hydrochloric acid and sodium hydroxide. After confirming the monodispersity of virus samples using particle size distribution measurements (described in section S2.1 in the supplemental material), viral zeta potentials were measured using electrophoretic light scattering measurements (Zetasizer Nano, Malvern Panalytical Ltd.), and the measurements were conducted at the Research Center for Environmental Quality Management at Kyoto University. For membranes, the zeta potential analyzer ELSZ-2000 (Otsuka Electronics Co., Ltd.) was used. The hydrophobic membrane (HB-0.45) was soaked in 50% v/v ethanol for 10 min, and then re-immersed in pure water for more than 1 h before measurement to facilitate water permeation. The same pretreatment was conducted before filtration tests of HB-0.45 to wet pores, and this method was modified by van Voorthuizen et al. (19).

### Virus and membrane contact angle measurements

The preparation of virus lawn membranes was described earlier (27). Briefly, purified virus stocks were filtered through a 50 kDa ultrafiltration membrane (polyethersulfone, Merck Millipore Ltd.) to form 5 layers of virions on the membrane surface. Virus-layered membranes were dried in air until the contact angle of water on virus lawns became a plateau value (28). The determination of plateau values was explained in section S2.2 in the supplemental material. Contact angles of surfaces were measured using the sessile droplet method with 2 µL DI water droplets. The entire process of dropping the droplet was filmed via a microscope (VHX-1000, KEYENCE), and then the shape of the droplet right after contacting a surface was captured for contact angle calculation.

### Virus adsorption tests on membranes

Virus adsorption to the membrane was investigated by the slightly modified method from Dika et al. (29). As adsorbents, membrane filters were used in this study. As test water, three purified viruses were added to the 200 mL of 10 mM NaCl at pH 7, and the virus concentration in the test water was 10^6^–10^8^ copies/mL (Table S4). Each membrane was cut to a diameter of 35 mm and soaked in test water for 30 min. This soaking duration was determined based on the longest filtration time in the next section. After removing membranes from the test water, water samples were collected as supernatant samples, and virus concentrations were measured. To confirm the adsorbed virus amount, membranes were soaked in 4 mL of lysis buffer (QIAGEN) for 15 min after being washed twice in 10 mM NaCl for 10 min. And then samples were collected as eluates from membranes. Virus amounts in test waters and eluates were quantified according to the protocol described below.

Virus cover rates on each membrane after the adsorption tests were calculated using the following equations:


(1) $$A_{virus}\;=\;\pi r_{virus}^{2}N$$

(2) $$virus\;cover\;rate\;\left(\%\right)=\frac{A_{MS2}+A_{PMMoV}+A_{AiV}}{A_{membrane}}\times 100$$

where *A*_virus_ is the total surface area covered by viruses (mm^2^/membrane), *r*_virus_ is the radius of the virus (half of the diameter listed in Table 1), *N* is the number of adsorbed viruses (copies/membrane), and *A*_membrane_ is the membrane surface area (1,920 mm^2^).

### Membrane filtration tests

As feed water, three purified viruses were added to the 10 mM NaCl solution at pH 7 to achieve 10^6^–10^7^ copies/mL. Lab-scale cross-flow filtration tests were conducted with a feed pump (FTU-1, Membrane Solution Technology) at the Research Center for Environmental Quality Management at Kyoto University. The filtration flux of each membrane was measured with 10 mM NaCl solution prior to the filtration test, and each condition is shown in Table 3. Membrane filtration tests were conducted more than three times, and 5 mL of feed and filtrate samples were collected to quantify virus concentrations. Filtrates were sampled three times in a single filtration run to confirm whether the virus concentration had changed. Filtration time with MF and UF membranes was within 5 min and 30 min, respectively. Filtration tests with the HB-0.45 membrane (hydrophobic MF) were conducted at two flow rates, as shown in Table 3.

**TABLE 3:** Filtration conditions

Membrane	Nominal pore size or MWCO	Pretreatment	Feed side pressure (kPa)	Cross-flow velocity (L/min)	Filtration flux (LMH, L/m^2^h)
HL-0.1	0.10 μm	No	70	1	1,960–2,040
HL-0.45	0.45 μm	No	70	1	4,240–6,110
HB-0.45	0.45 μm	Yes	70	1/2	5,620–6,680/ 5,700–8,150
UF-1k	1 kDa	No	250	1	33–63

### Virus quantification and calculation of log removal values

Viruses in samples were quantified by real-time quantitative PCR (qPCR) assay. For viral RNA extraction, the QIAamp Viral RNA Mini Kit (QIAGEN) was used, and 60 μL of extract was obtained from 140 μL of each sample. Right after RNA extraction steps, reverse transcription steps were conducted with a high-capacity cDNA reverse transcription kit (Applied Biosystems). A portion (5 μL) of cDNA was mixed with PrimeTime Gene Expression Master Mix (Integrated DNA Technologies), primers, probe, and distilled water. Primers and probe sequences were reported in previous studies (30–32). In each PCR run, negative control wells were included and no amplification was observed. The concentrations of Ct = 40 were described as the detection limit. Log removal values (LRVs) were calculated with the following formula:


(3) $$LRV=\log_{10}\left(\frac{C_{0}}{C}\right)$$

where *C*_0_ is the initial virus concentration in feed water and *C* is the virus concentration in filtrates or supernatants.

## RESULTS

### Virus surface properties

**Fig 1:**
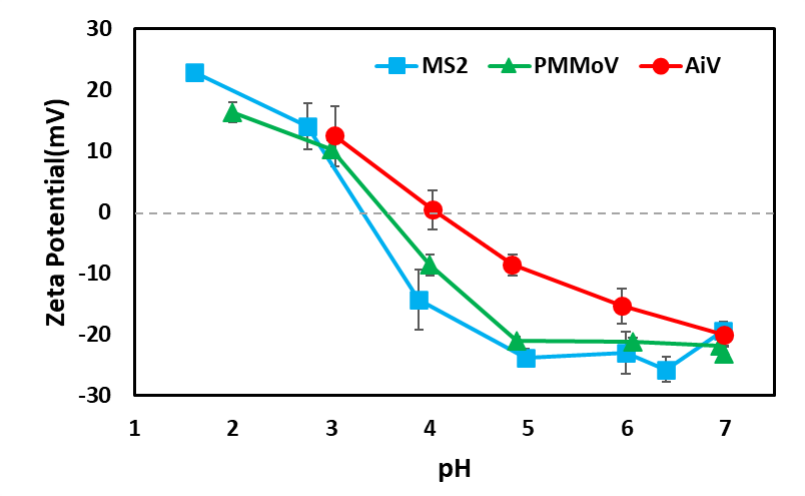
Zeta potentials of viruses as a function of pH.

In this section, the zeta potentials and contact angles of the three viruses, MS2, PMMoV, and AiV, were measured to characterize their surface properties. Table 4 summarizes the measured zeta potentials at pH 7, isoelectric points (pI), and contact angles for each virus. Fig. 1 shows the measured zeta potentials of viruses as a function of pH. All viruses exhibited low isoelectric points (pIs): 3.3 for MS2, 3.5 for PMMoV, and 4.1 for AiV. These values are consistent with those reported in previous studies: 3.1–3.9 for MS2, 3.2–3.8 for PMMoV, and 3.5 for AiV (17, 22, 33–35). Viral zeta potentials at pH 7 ranged from −19.4 to −23.2 mV, showing minimal variation among viruses (Table 4). On the other hand, the measured contact angles varied among viruses, indicating differences in surface hydrophobicity. As a higher contact angle corresponds to greater hydrophobicity, AiV exhibited the highest degree of hydrophobicity, followed by PMMoV and MS2, with the trend: MS2 ≤ PMMoV < AiV.

**TABLE 4:** Zeta potentials at pH 7 and contact angles of viruses

Parameter	MS2	PMMoV	AiV
Zeta potential (mV)	−19.4 ± 1.6	−23.2 ± 1.2	−20.1 ± 1.7
Isoelectric point	3.3	3.5	4.1
Contact angle (°)	48.7 ± 2.3	51.7 ± 8.1	72.4 ± 4.8

### Membrane surface properties

The zeta potentials and contact angles of the four membranes used in the adsorption and membrane filtration tests were measured. Table 5 summarizes zeta potentials at pH 7 and water contact angles of the membranes. All membranes exhibited negative charges at neutral pH, with zeta potentials ranging from −12.2 to −22.8 mV. Among all the membranes, HB-0.45, a hydrophobic MF membrane, was the most negatively charged, and HL-0.45, a hydrophilic MF membrane, was the least. The contact angle of HB-0.45 was 118°, indicating a highly hydrophobic surface. In contrast, HL-0.1, HL-0.45, and UF-1k exhibited relatively hydrophilic surfaces. The hydrophobicity followed the order: HL-0.1 < HL-0.45 < UF-1k < HB-0.45.

**TABLE 5:** Zeta potentials at pH 7 and contact angles of membranes

Parameter	HL-0.1	HL-0.45	HB-0.45	UF-1k
Zeta potential (mV)	−19.9 ± 5.9	−12.2 ± 1.3	−22.8 ± 1.0	−19.4 ± 11.7
Contact angle (°)	60.7 ± 2.3	73.7 ± 3.0	118.0 ± 2.1	83.0 ± 1.1

### Virus adsorption on membranes

We evaluated the virus adsorption capacity of the membranes under non-flow conditions. The LRVs, calculated using initial and supernatant virus concentrations based on equation 3, ranged from −0.3 to 0.8 (Table S5). Negative LRVs indicate that the virus filtrate concentration was slightly higher than the initial concentration. This is due to the limitations of the qPCR assay, which cannot reliably distinguish differences smaller than tenfold. These near-zero LRVs indicate that most viruses were not adsorbed onto the membranes and remained in the aqueous phase under conditions without water flow.

**Fig 2:**
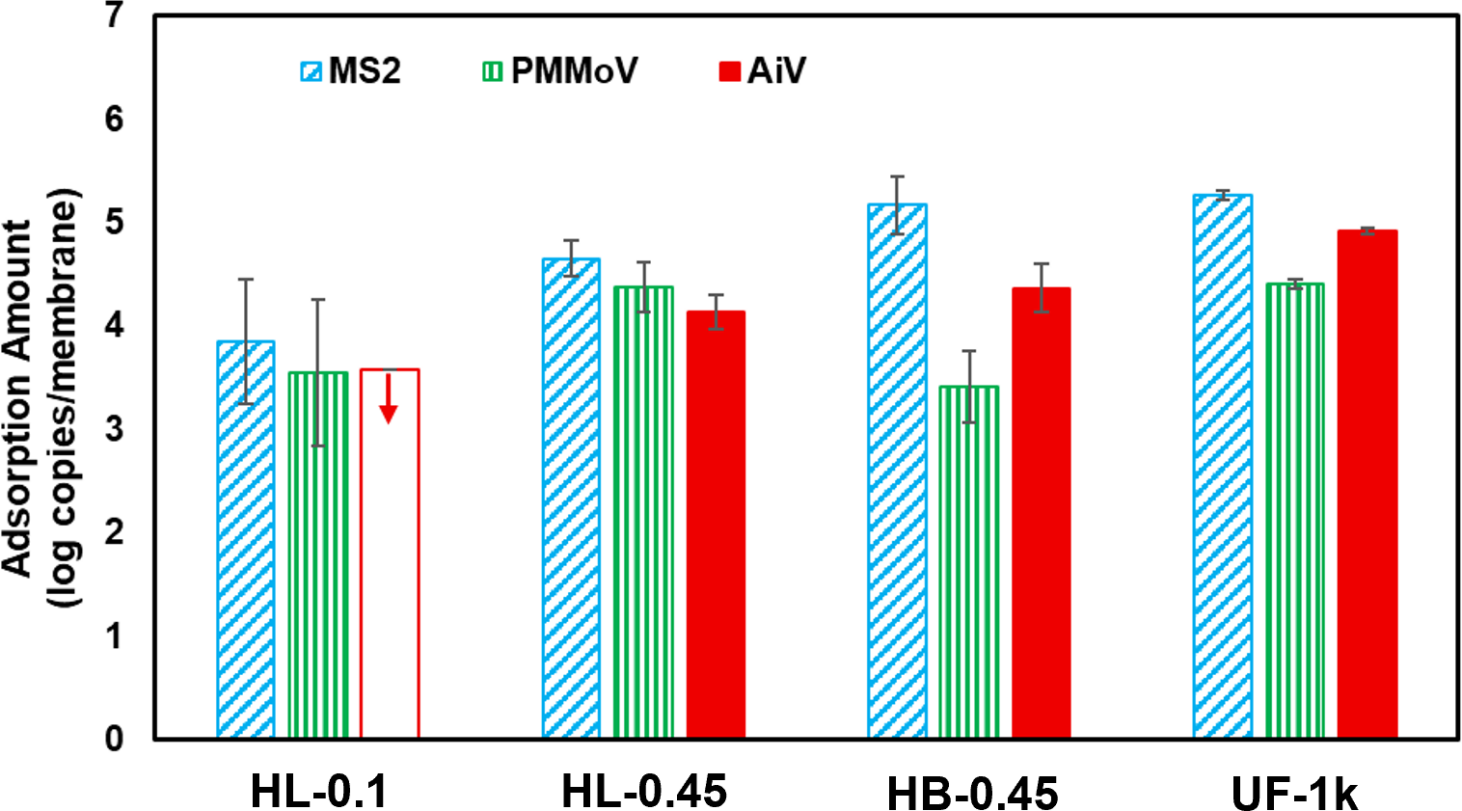
Virus adsorption amount of each membrane. The downward arrow indicates that the virus concentration was below the detection limit.

The amounts of viruses adsorbed onto each membrane are shown in Fig. 2. The highest observed adsorption was 5.3 log copies/membrane of MS2 on the UF-1k membrane. Virus cover rates on each membrane were calculated with equations 1 and 2 and are shown in Table 6. The maximum surface coverage was only 15.2 × 10⁻⁶% of the membrane area. Therefore, this adsorption was not reflected in LRVs. The LRVs calculated by the adsorption amount on membranes were zero for all cases. Consistent with this, the virus concentration in the post-adsorption liquid remained unchanged at 7.5 log copies/mL, the same as the initial concentration (Table S4). Among viruses, the amount of PMMoV adsorbed onto the HB-0.45 and UF-1k membranes was significantly smaller than that of MS2 and AiV (one-sided *t*-test, *P* < 0.05, Fig. 2).

**TABLE 6:** Total surface area covered by viruses and virus cover rates on each membrane surface

Parameter	HL-0.1	HL-0.45	HB-0.45	UF-1k
MS2 (×10^−6^ mm^2^/membrane)	5.8 ± 4.5	24.8 ± 9.0	89 ± 56	96 ± 11
PMMoV (×10^−6^ mm^2^/membrane)	34 ± 28	142 ± 69	17 ± 12	137 ± 15
AiV (×10^−6^ mm^2^/membrane)	–^*a*^	10.1 ± 4.2	18 ± 10	58.1 ± 4.2
Cover rate (×10^−6^%)	–	9.2 ± 4.1	6.4 ± 3.5	15.2 ± 0.6

^*a*^ –, AiV on HL-0.1 was not detected.

### Virus LRVs by membrane filtration

**Fig 3:**
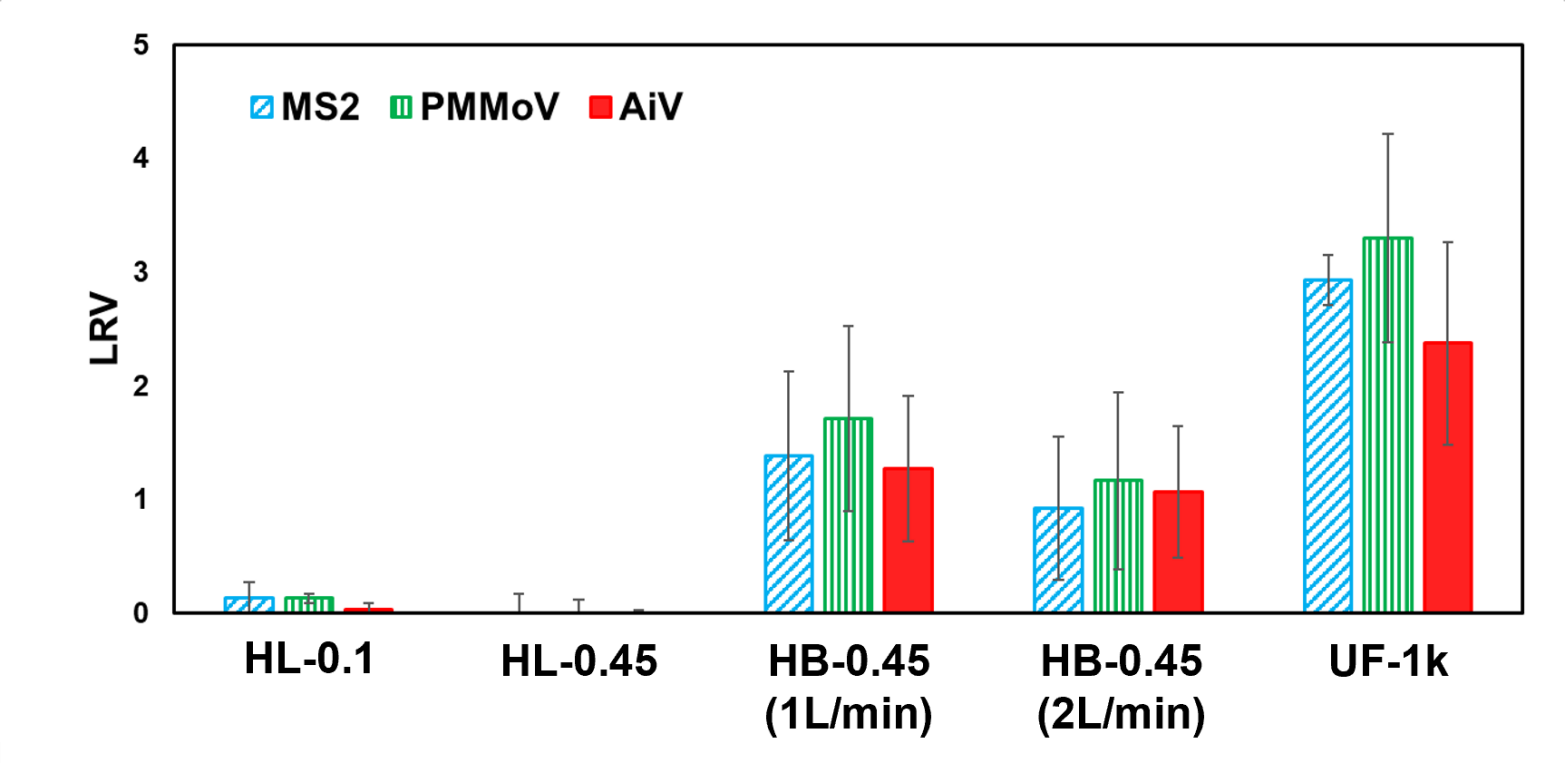
Average virus LRVs by membranes.

Membrane filtration tests with the four membranes were performed, and virus removal capacity was investigated. Virus LRVs of the membranes calculated with equation 3 are shown in Fig. 3. LRVs of MF were calculated based on the average concentration from triplicate filtrate samples, and those of UF were calculated based only on concentrations of filtrates from the first 10 min to avoid a time-dependent decrease in concentrations (Table S7). Virus removal was observed for the HB-0.45 (hydrophobic MF membrane) and UF-1k, while no removal was observed for the HL-0.45 membrane (hydrophilic MF). LRVs for the HB-0.45 membrane were 1.4 and 0.9 for MS2, 1.7 and 1.2 for PMMoV, and 1.3 and 1.1 for AiV under low (1 L/min) and high cross-flow velocities (2 L/min), respectively. Average LRVs by HB-0.45 membrane with low flux were slightly higher than those with high flux, although significant differences were not observed because of the high variation in LRVs.

UF-1k exhibited the highest LRVs among all membranes. The average LRVs were 2.9 for MS2, 3.3 for PMMoV, and 2.4 for AiV. In one trial, AiV was not detected in the UF-1k filtrate; therefore, its average LRV was calculated using only samples in which viruses were detected (Table S8). The molecular weight cutoff of UF-1k was 1 kDa, which is smaller than all target viruses, indicating that virus removal occurred via size exclusion.

The average LRVs by the HL-0.1 and HL-0.45 membranes were 0.1 and −0.1 for MS2, 0.1 and −0.1 for PMMoV, and 0.0 and −0.1 for AiV, respectively. The negative LRVs indicate slightly higher virus concentrations in filtrates than in feed water, likely due to inherent quantification errors in qPCR as mentioned in the previous section.

## DISCUSSION

### Virus and membrane surface properties

The viruses and membranes were all negatively charged under neutral pH conditions (Tables 4 and 5). Our results indicate that electrostatic repulsions would occur between viruses and membranes during the adsorption and filtration tests. Consequently, viruses must overcome these electrostatic repulsive forces to attach to membrane surfaces.

The measured zeta potentials and calculated pIs of MS2, PMMoV, and AiV were similar, ranging from −19.4 to −23.1 mV and 3.3 to 4.1, respectively (Table 4). The charge characteristics observed here are consistent with those reported previously (34, 35). This similarity suggests that the strength of electrostatic repulsion among the three viruses was nearly equivalent, implying that their charge characteristics are unlikely to make their removal behavior significantly different.

In contrast, contact angles, which represent hydrophobicity, were different among the viruses although they are below 90° (Table 4). Based on the average values, hydrophobicity followed the order MS2 < PMMoV < AiV, with MS2 and PMMoV exhibiting similar values (48.7° vs. 51.7°). AiV showed the highest hydrophobicity, suggesting that it may be the most adsorbed onto or removed by membranes among the three viruses.

Although studies evaluating hydrophobicity of MS2, PMMoV, and AiV are limited, MS2 has been assessed using various approaches (e.g., contact angle measurements, adsorption tests, and structure-based modeling) (34, 35). In most cases, MS2 exhibited relatively lower hydrophobicity among the target viruses (18, 22, 23), consistent with our findings. However, reported contact angles for MS2 varied widely, ranging from 28° to 84° (18, 33, 36, 37), and Attinti et al. (33) reported a smaller contact angle of AiV than for MS2 (28° vs. 33°), which contrasts with our findings (33). Although the contact angle method enables quantitative evaluation of hydrophobicity, it is influenced by surface heterogeneity and roughness (38). Such heterogeneity may arise from the presence of mutant strains generated during cultivation or from structural alterations due to viral inactivation during drying. A major difference between this study and previous research lies in the purification methods employed, which may account for the observed discrepancies. In the present study, we employed a two-step ultracentrifugation process involving both sucrose and CsCl density gradient separations to obtain high-purity AiV stock solutions (Fig. S2). Shi et al. (39) demonstrated that the remaining impurities after PEG precipitation and centrifugal diafiltration changed the particle size distributions, zeta potentials, and contact angles of viruses (39). Therefore, in this study, density gradient centrifugation using sucrose and CsCl was employed for purification without PEG precipitation. The AiV used in this study showed a particle distribution with a 31.2 nm peak, close to the reference value (Fig. S2; Table S1). Therefore, the measured contact angles are considered to represent the intrinsic surface properties of the virus particles themselves.

### Virus adsorption onto membranes

In the adsorption test, the reduction calculated from the initial and supernatant virus concentrations ranged from −0.3 to 0.8 log (Table 6), indicating that viruses were not reduced by more than a factor of 10. On the other hand, eluted virus amounts from membranes ranged from 3.4 to 5.3 log copies/membrane (Fig. 3). This is between one in 100,000 and one in 1,000,000 of the virus load in the test water. Based on virus cover rates, the maximum surface coverage was only 15.2 × 10⁻⁶%, indicating that most of the membrane surface remained unoccupied by viruses. These results suggest that the attractive interactions between viruses and the membrane are insufficient to promote significant adsorption under static conditions. Electrostatic repulsion between viruses and membranes was expected, as both exhibited negative charges at pH 7 (Tables 4 and 5), which may have inhibited virus adsorption onto the membranes. To overcome this repulsion, energy is required to bring viruses into close proximity to the membrane surface. During membrane filtration, hydrodynamic forces may facilitate this transport, thereby enhancing virus adsorption.

Although the amounts of viruses adsorbed onto the membranes were small, PMMoV exhibited lower adsorption to the HB-0.45 and UF-1k membranes than MS2 and AiV (Fig. 3). This may be due to lower initial PMMoV concentrations than those of the others (Table S4). The other possible explanation for this difference is its lower diffusivity than others (Table S1). In the absence of flow, contact between viruses and membranes may be primarily driven by Brownian motion. Diffusion coefficients and diffusion distances of viruses in 30 min are shown in Table S1. Because PMMoV is substantially larger than MS2 and AiV (Table 1), the diffusion distance of PMMoV was almost half of those of MS2 and AiV (Table S1). PMMoV may exhibit lower mobility in water, resulting in a lower collision frequency with the membrane surface.

### Virus removal by membranes

Compared to the adsorption tests, high removal values were observed in the filtration tests (Table 6 vs Fig. 3). The filtration with UF-1k membrane exhibited the highest LRVs; however, viruses were detected in filtrates (Fig. 3), even though the UF-1k membrane has smaller pores than viruses. The reason for virus breakthrough from the UF membrane might be large pores within the pore size distribution (40). Although HB-0.45 membranes showed less than one log removal in the adsorption tests, they achieved at most 2.4-log removal by the filtration tests (Table 6; Table S8). This enhanced adsorption might be attributed to increased opportunities for viruses to contact the HB-0.45 membrane, as viruses pass through the membrane interior during filtration.

LRVs of viruses ranging from 0.9 to 1.7 by the HB-0.45 membrane (Table S8) suggest that hydrophobic-driven interaction contributed to virus removal even in the absence of size exclusion. Yasui et al. (18) also reported higher LRVs by a hydrophobic MF membrane in high ionic strength conditions than those in low ionic strength ones because electrostatic repulsion was mitigated (18). However, in our study, the electrostatic repulsion between viruses and the membrane surface was expected because of their negative zeta potentials (−22.8 mV for HB-0.45 and approx. −20 mV for virions, Tables 4 and 5). Nevertheless, viruses were removed, indicating that viruses can overcome these repulsive interactions. Hashimoto et al. (41) reported that Brownian force can enhance nanoparticle retention within pores by inducing collisions with membranes (41). In our study, the Brownian force may have brought viruses close enough to the pore surface to overcome electrostatic repulsion and facilitate their attachment. It should also be noted that virus surfaces have heterogeneous charge distributions. Armanious and Mezzenga (42) suggested that, since virus–surface interactions occur over a limited contact area, localized positively charged regions on the virus surface may promote adsorption (42). Similar heterogeneity likely exists in surface hydrophobicity and may further influence virus adsorption following transport to the membrane surface via Brownian motion. Further analysis of viral surface properties is required to clarify these mechanisms.

The LRV decrease with increasing filtration flux (Fig. 3) suggests that high filtration flux may inhibit the virus diffusion in membrane pores. Inhibited diffusion may decrease the opportunities for viruses to collide with pore surfaces, thereby causing less adsorption of viruses to the HB-0.45 membrane. On the contrary, Trilisky and Lenhoff (43) reported greater virus retention with increasing flow rates under conditions where viruses did not interact with a filtration device (43). Similarly, Yamamoto et al. (44) (2014) mentioned three critical mechanisms of virus removal by membranes: size exclusion, constraint by hydrodynamic forces, and multistep filtration. Both studies concluded that particle diffusion is suppressed at high flow rates, thereby preventing leakage through relatively large pores in the filtration system. Although these studies assumed size exclusion and thus differed in premise from the present study, they suggest that diffusion is affected by filtration flux. Without size exclusion, high filtration flux may mitigate virus adsorption.

### Promising performance indicator viruses

Among the tested viruses, PMMoV exhibited the highest LRV by membrane filtration tests (Fig. 3). Three viruses showed similar zeta potentials, and PMMoV’s contact angle was intermediate among the three viruses. Therefore, the highest LRV is likely due to PMMoV’s morphology. During the filtration, viruses passed through the membrane pores, and PMMoV’s larger, rod-shaped structure compared with the spherical MS2 and AiV may have led to entrapment within the membrane’s complex pore structure. Depending on the membrane material, pore structures may become more complex, potentially leading to higher removal of viruses with morphologies similar to that of PMMoV.

From the perspective of selecting a resilient performance indicator for membrane filtration, spherical viruses such as MS2 and AiV appear to be more suitable than rod-shaped viruses such as PMMoV. MS2 and AiV did not exhibit a significant difference in their LRVs. Although AiV was expected to exhibit higher removal due to its greater hydrophobicity (its contact angle of 72.4°, larger than that of MS2), this did not result in enhanced LRVs. These findings suggest that virus removal values obtained using MS2 may be comparable to those obtained using AiV.

An important consideration is that the present experiments focused solely on virus–membrane interactions. In actual treatment systems, water chemistry is more complex and may alter their removal behavior. Under such conditions, viruses’ LRVs may differ from those observed in this study. In the case of studies using environmental water as test water, PMMoV has not always shown the higher LRVs than spherical viruses (45, 46). Furthermore, viruses may not be monodispersed in environmental waters, as used in this study. Viruses may associate with suspended solids or other particles and form aggregates. Wu et al. (47) reported that when secondary-treated wastewater was fractionated using a membrane, RNA viruses, including PMMoV, were most abundant in the fraction that passed through the smallest pore size (0.45 µm), whereas DNA viruses were more frequently associated with larger particles (47). Since larger particles are more readily removed by the membrane, the RNA viruses examined in this study may be more suitable as indicator viruses than DNA viruses. Moreover, if these viruses are less prone to aggregation, the evaluation results obtained using the monodisperse viruses in this study may better reflect removal trends in full-scale water treatment plants. Although further investigation is required to clarify virus aggregation behavior in environmental waters, our findings highlight the important role of viral morphology in removal mechanisms during membrane filtration. In this context, spherical viruses may serve as more resilient performance indicators than rod-shaped viruses.

In addition, membrane filtration combined with pre-coagulation or coagulation-sedimentation has sometimes exhibited lower LRVs of PMMoV than those of other viruses (45), in contrast to the present findings. Virus LRVs during coagulation have been reported to increase in the order of virus hydrophobicity, indicating that more hydrophobic viruses coagulate more readily (48, 49). Therefore, after coagulation processes, virus hydrophobicity may exert a more pronounced influence on virus removal than size or morphology. It should also be noted that more hydrophilic viruses than MS2 may exhibit lower LRVs than MS2 and AiV. Therefore, when selecting a resilient indicator relative to pathogenic viruses, the hydrophilicity of pathogenic viruses should be evaluated. However, the surface properties of pathogenic viruses, such as Norovirus and Rotavirus, are still unknown, and further study is required to reveal this point.

### Conclusions

The surface properties of three viruses—performance indicator virus candidate MS2, PMMoV, and AiV—were experimentally measured, and virus adsorption and filtration tests with membranes were conducted. Although viruses’ zeta potentials at pH 7 in 10 mM sodium chloride solution were similar, ranging from −19.4 to −23.2 mV, their contact angles varied from 48.7° to 72.4°, indicating differences in hydrophobicity in the order: MS2 < PMMoV < AiV.

Adsorption and filtration tests using hydrophobic and hydrophilic membranes demonstrated that viruses were removed by the hydrophobic membrane, even under electrostatically repulsive conditions. Our results indicate that virus diffusion during filtration can overcome electrostatic repulsion, facilitating collisions with the pore surfaces.

However, the amounts of viruses adsorbed onto and removed by membranes were not correlated with the viruses’ hydrophobicity. Among the tested viruses, PMMoV exhibited the highest LRVs during membrane filtration, likely due to its rod-shaped structure. In contrast, the LRVs of AiV and MS2—the most and least hydrophobic viruses, respectively—did not differ significantly. This result suggests that LRVs of MS2 and AiV by membrane filtration are comparable despite their different surface properties. Although the effects of water chemistry and virus association with suspended solids need to be investigated, our results suggest that spherical viruses, such as MS2 and AiV, may serve as more resilient performance indicators for membrane filtration than rod-shaped viruses such as PMMoV.

## Data Availability

qPCR and other data obtained in this study are available upon request.

## SUPPLEMENTAL MATERIAL

Supplemental material aem.00035-26-s0001.docx Supplemental methods, Fig. S1 and S2, and Tables S1 to S8.

ASM does not own the copyrights to Supplemental Material that may be linked to, or accessed through, an article. The authors have granted ASM a non-exclusive, world-wide license to publish the Supplemental Material files. Please contact the corresponding author directly for reuse.
