# Supplementary material for: Resilient performance indicator virus for membrane filtration considering surface charge, hydrophobicity, and morphology
Source: Appl Environ Microbiol. 2026 May 11;92(6):e00035-26. doi: 10.1128/aem.00035-26 (PMC13274348; doi:10.1128/aem.00035-26)
Supplement: Supplemental material — Supplemental methods, Fig. S1 and S2, and Tables S1 to S8. [file aem.00035-26-s0001.docx]

Resilient performance indicator virus for membrane filtration considering surface charge, hydrophobicity and morphology

Midori Yasui^1)*^, Tatsuya Sakai^1)^, Takashi Hashimoto^2)^, Sadahiko Itoh^1)^

1) Department of Environmental Engineering, Kyoto University

2) Department of Urban Engineering, the University of Tokyo

*Corresponding author: yasui.midori.5f@kyoto-u.ac.jp

1. Virus Propagation and Quantification
   1. Propagation of Pepper Mild Mottle Virus (PMMoV)

The seeds were grown in seedling pods filled with gardening soil and cultured at 25 °C under a long-day photoperiod (16-h light, 8-h dark) and then the seedlings were transplanted in individual pods with fresh garden soil. The seedlings were grown for approx. 2 weeks until obtaining the 5-6 leaf stage plants. Milli-Q water was added to the pods every 2 days during cultivation.

PMMoV strains were obtained from the National Agriculture and Food Research Organization (NARO). After freezing infected plant tissues, they were crushed and added to a phosphate buffer to extract viruses. The solution with plant tissues was centrifuged (6,000 g, 10 min) and filtered by cellulose acetate membrane (pore size 0.2 μm, ADVANTEC). The filtrate was stored at 4 °C until use as the stock solution.

After host plant cultivation, one of the leaves from each pod was scratched with 600-mesh carborundum, and then 100 μl of PMMoV stock solution was applied to inoculate the plant with PMMoV. The carborundum on the leaf was washed away with Milli-Q water. The inoculated plants were cultivated at 25 °C under a long-day photoperiod for approximately. 1 week. After that, infected leaves were removed and viruses were extracted, as mentioned previously.

- 1. Propagation of Aichi Virus (AiV)

As host cells, the Vero cell line (JCRB9013), obtained from the National Institute of Biochemical Innovation, was used. The Vero cells at 90 % of confluence were inoculated with AiV, provided by Prof. Hiroyuki Katayama, the University of Tokyo, in a cell culture flask (25 cm^2^) and incubated in 37 °C and 5% CO2 for 2-3 days in a CO2 incubator. After propagation, the 3 times freeze-thaw steps were conducted and then the suspension was centrifuged (8,000 g, 10 min). The supernatant was passed through a cellulose acetate membrane (pore size 0.2 μm, ADVANTEC) and stored at 4 °C until use as the stock solution.

- 1. Plaque assay to quantify infectious MS2

Before and after the purification process, MS2 concentrations were measured using the single-layer agar method with E. coli K12 F+ A/λ(NBRC3301) as the host strain. Samples were mixed with log-phase host culture and LB agar, and then poured into Petri plates. Plates were incubated at 37 °C overnight, and emerging plaques were counted. A significant loss of MS2 was not observed after purification processes.

1. Virus surface property measurement
   1. Purity and particle number in virus stocks

After propagation, virus samples were purified by ultracentrifugation. Figure S1 shows the estimated particle concentration in samples. They were measured by Zetasizer Ultra (Malvern Panalytical Ltd.). MS2 and PMMoV stocks showed monodisperse particles of one size after CsCl ultracentrifugation. However, AiV showed multiple particle peaks after one-step CsCl ultracentrifugation (Fig. S1C), arising from host debris. After two-step ultracentrifugation, a mono-peak was obtained (Fig. S1D). In this study, a two-step purification was used for AiV.

| (A) | (B) |
| --- | --- |
| 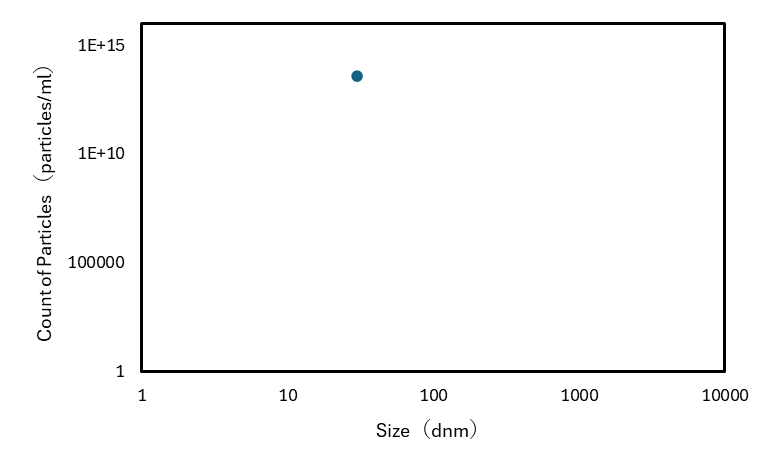 | 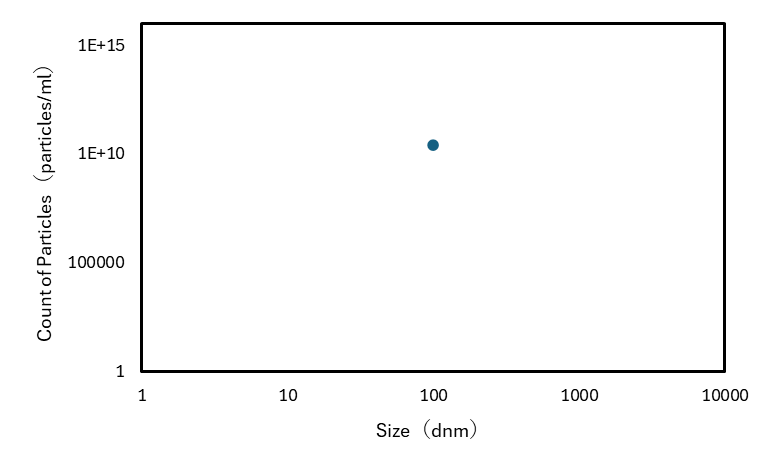 |
| (C) | (D) |
| 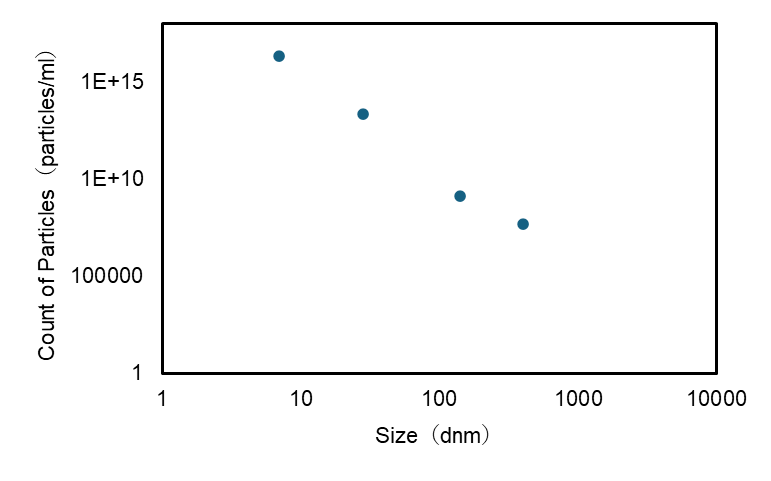 | 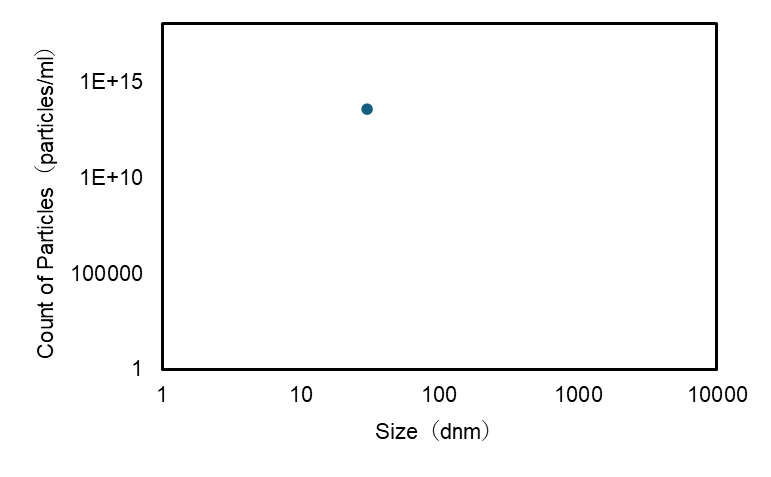 |

Figure S1. Particle sizes and concentrations after ultracentrifugation

A: MS2, B: PMMoV, C: AiV (one-step), D: AiV (two-step)

- 1. Evaluation of “plateau values” of target virus contact angles

The virus cake layer was dried in air until the surface contact angles remained stable values for 30 minutes to 1 hour. This status is described as the plateau value (van Oss, 2006). The timing of the “plateau value” of the virus-cake membrane surface was determined by measuring the contact angles of MilliQ water as a function of the drying time. Results are shown in Figure S1. The virus cake membranes were prepared with a 50 k UF membrane (PES, Merck Millipore Ltd.). The timing at which the water contact angle reached its plateau value (shown as red squares in Fig. S1) was confirmed before each measurement.

| (A) | (B) |
| --- | --- |
| 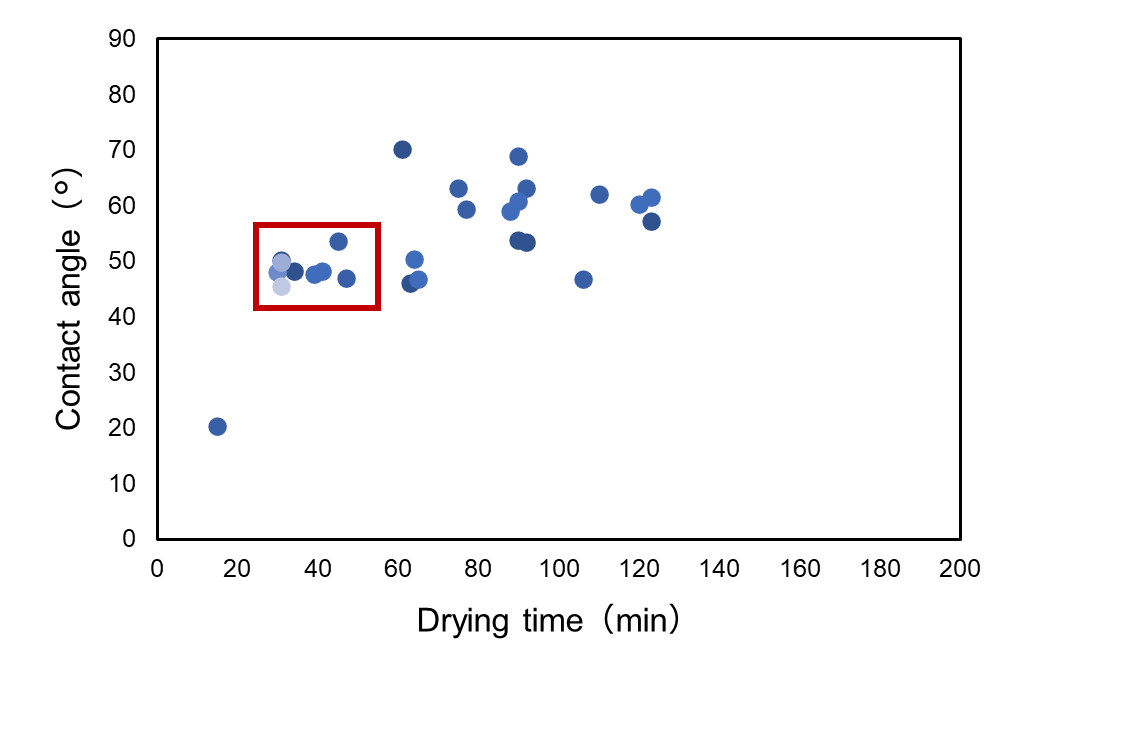 | 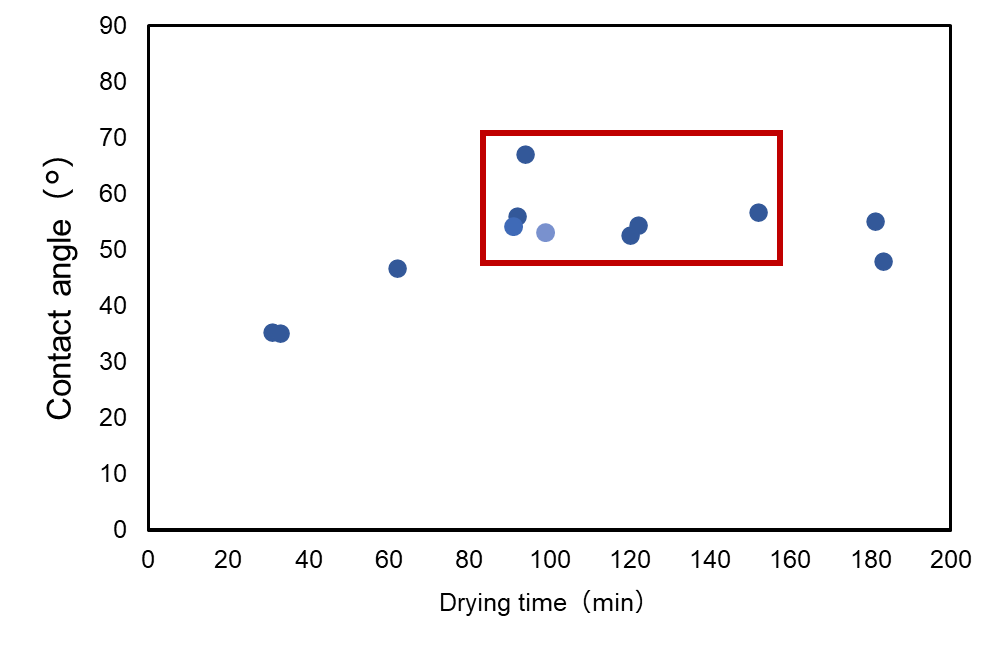 |
| (C) |  |
| 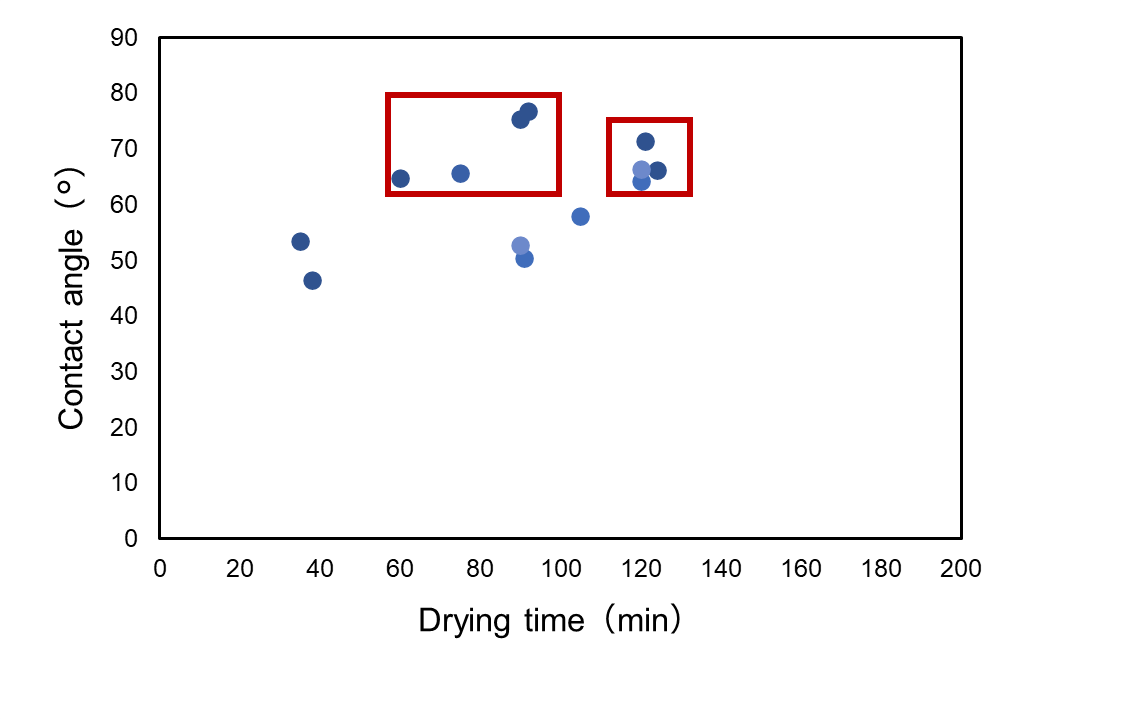 |  |

Figure S2. Contact angles of virus surfaces as a function of drying time

A: MS2, B: PMMoV, C: AiV

*The different colors of the plots indicate that they are different membranes.

- 1. Virus particle size and diffusion coefficients

Virus particle sizes in samples were measured before zeta potential measurement by the dynamic light scattering (DLS) method (Zetasizer Nano, Malvern Panalytical Ltd.). The diffusion coefficient of each virus was calculated using the Stokes–Einstein equation:

$$D_{p}=\frac{k_{B}T}{3\pi\eta a}$$

$k_{B}$ is Boltmann constant, 1.380649×10^-23^, $T$ is temperature, and $\eta$ is water viscosity, 0.890×10^−3^ Pa·s at 25 °C. $a$ is the particle diameter, using the measured virus size. The diffusion distance in 30 min was calculated using the following formula:

$$l=\sqrt{6D_{p}t}$$

Table S1 summarizes the measured virus sizes and calculated values. Among three viruses, PMMoV exhibited the largest particle size. PMMoV has a rod-shaped morphology, with approx.18 nm in diameter and 300 nm in length. Although its diameter is smaller than MS2 and AiV (approx. 30 nm), its length is roughly ten times greater. DLS measurements of rod-shaped particles are generally influenced by rotational motion, which reflects their length rather than diameter. The measured particle size of PMMoV was 88 nm, smaller than its length, suggesting the possibility that some virions were in a shortened or curled state during measurement.

The diffusion coefficient represents the ease of particle diffusion. Among three viruses, PMMoV showed the highest diffusion coefficient and the least movement during the adsorption and filtration tests due to the relatively large particle size.

Table S1. Virus sizes, diffusion coefficients, and diffusion distances in 30 min

|  | Virus size (nm) | Diffusion coefficient (m^2^/s) | Diffusion distance (µm) |
| --- | --- | --- | --- |
| MS2 | 33.2±8.0 | $1.62\times{10}^{-11}$ | 41.9 |
| PMMoV | 88.2±1.3 | $5.56\times{10}^{-12}$ | 24.5 |
| AiV | 30.2±7.4 | $1.48\times{10}^{-11}$ | 39.9 |

- 1. Zeta potentials and contact angles of viruses and membranes

Table S2 shows all the measured zeta potentials of viruses and membranes. Table S3 shows all the measured contact angles of viruses and membranes. Their average values are shown in Tables 2 and 3.

Table S2. Virus and membrane zeta potentials

| Target | Zeta potential (mV) | | | | |
| --- | --- | --- | --- | --- | --- |
| MS2 pH1.6 | 21.6 | 22.6 | 23.9 | 23.0 |  |
| MS2 pH2.8 | 17.0 | 16.4 | 7.8 | 15.7 | 13.0 |
| MS2 pH3.9 | -7.8 | -14.5 | -18.1 | -19.7 | -11.2 |
| MS2 pH5.0 | -23.5 | -24.4 | -21.8 | -25.4 |  |
| MS2 pH6.0 | -24.1 | -19.7 | -27.4 | -20.9 |  |
| MS2 pH6.4 | -24.6 | -24.1 | -28.7 | -25.4 |  |
| MS2 pH7.0 | -19.0 | -21.2 | -18.1 |  |  |
| PMMoV pH2.0 | 18.1 | 15.1 | 15.6 |  |  |
| PMMoV pH3.0 | 11.1 | 11.1 | 8.7 |  |  |
| PMMoV pH4.0 | -7.4 | -8.0 | -10.6 |  |  |
| PMMoV pH4.9 | -21.4 | -21.2 | -20.3 |  |  |
| PMMoV pH6.1 | -21.5 | -21.5 | -20.4 |  |  |
| PMMoV pH6.95 | -21.8 | -20.9 | -23.0 |  |  |
| PMMoV pH6.99 | -23.2 | -22.3 | -24.5 | -22.6 |  |
| AiV pH3.0 | 15.9 | 8.9 |  |  |  |
| AiV pH4.0 | 3.2 | 1.0 | -3.2 |  |  |
| AiV pH4.8 | -7.4 | -8.0 | -10.6 |  |  |
| AiV pH6.0 | -12.3 | -15.6 | -18.0 |  |  |
| AiV pH7.0 | -18.5 | -21.0 | -22.0 | -18.9 |  |
| VVLP pH 7.0 | -14.9 | -18.5 | -26.4 |  |  |
| HVLP pH 7.0 | -12.1 | -13.6 | -11.0 |  |  |
| HVHP pH 7.0 | -22.3 | -24.0 | -22.2 |  |  |
| UF-PES pH 7.0 | -13.0 | -36.6 | -11.4 | -16.7 |  |

Table S3. Virus and membrane contact angles.

| Target | Contact angle (°) | | | | |
| --- | --- | --- | --- | --- | --- |
| MS2 | 50.2 | 48.2 | 53.5 | 46.9 | 47.8 |
|  | 48.2 | 48.1 | 49.8 | 45.4 |  |
| PMMoV | 56.0 | 67.1 | 52.6 | 54.4 | 54.2 |
|  | 53.0 | 44.6 | 42.0 | 41.1 |  |
| AiV | 75.3 | 76.9 | 71.5 | 66.1 |  |
| HL-0.1 | 59.0 | 58.7 | 63.6 | 61.5 |  |
| HL-0.45 | 75.3 | 75.3 | 74.9 | 69.2 |  |
| HB-0.45 | 117.7 | 121.3 | 115.7 | 118.3 | 117.0 |
| UF-1k | 82.1 | 83.4 | 84.4 | 82.2 |  |

1. Virus adsorption on membranes

Table S4 summarizes virus concentration in bulk water, post-adsorption solution and eluate. Each experiment was conducted three times, and the average concentrations are shown. Post-adsorption 1 and 2 were collected from the center and edge of the container, respectively. Table S5 shows LRVs calculated from the initial and post-adsorption concentrations.

Table S4. Average virus concentrations in bulk water/post-adsorption solution/eluate (log copies/ml or membrane)

| Membrane | Virus | Initial | Post-adsorption 1 | Post-adsorption 2 | Eluate (/membrane) |
| --- | --- | --- | --- | --- | --- |
| HL-0.1 | MS2 | $6.8\pm1.7$ | $6.5\pm0.8$ | $6.7\pm1.0$ | $3.8\pm0.6$ |
|  | PMMoV | $6.1\pm1.8$ | $6.5\pm1.5$ | $6.4\pm1.5$ | $3.5\pm0.7$ |
|  | AiV | $6.2\pm0.6$ | $6.4\pm0.7$ | $6.5\pm0.4$ | $3.6\pm0.0$ |
| HL-0.45 | MS2 | $7.9\pm1.0$ | $7.1\pm0.1$ | $7.3\pm0.4$ | $4.6\pm0.2$ |
|  | PMMoV | $7.3\pm0.2$ | $7.1\pm0.2$ | $7.3\pm0.1$ | $4.4\pm0.2$ |
|  | AiV | $6.8\pm0.1$ | $6.7\pm0.0$ | $6.7\pm0.2$ | $4.1\pm0.2$ |
| HB-0.45 | MS2 | $7.4\pm0.2$ | $6.6\pm0.4$ | $6.6\pm0.5$ | $5.1\pm0.3$ |
|  | PMMoV | $6.3\pm0.1$ | $6.3\pm0.1$ | $6.3\pm0.0$ | $3.4\pm0.0$ |
|  | AiV | $6.7\pm0.1$ | $6.7\pm0.1$ | $6.7\pm0.0$ | $4.4\pm0.2$ |
| UF-1k | MS2 | $7.5\pm0.3$ | $7.5\pm0.3$ | $7.6\pm0.2$ | $5.3\pm0.0$ |
|  | PMMoV | $6.3\pm0.0$ | $6.4\pm0.0$ | $6.4\pm0.0$ | $4.4\pm0.0$ |
|  | AiV | $6.8\pm0.0$ | $6.8\pm0.1$ | $6.9\pm0.1$ | $4.9\pm0.0$ |

Table S5. Average virus LRVs by adsorption tests

| Membrane | Virus | LRV 1 | LRV 2 |
| --- | --- | --- | --- |
| HL-0.1 | MS2 | 0.28 | 0.12 |
|  | PMMoV | -0.29 | -0.30 |
|  | AiV | -0.17 | -0.29 |
| HL-0.45 | MS2 | 0.77 | 0.58 |
|  | PMMoV | 0.16 | 0.01 |
|  | AiV | 0.11 | 0.06 |
| HB-0.45 | MS2 | 0.80 | 0.81 |
|  | PMMoV | 0.03 | 0.03 |
|  | AiV | 0.04 | 0.01 |
| UF-1k | MS2 | -0.01 | -0.08 |
|  | PMMoV | -0.02 | -0.05 |
|  | AiV | -0.05 | -0.10 |

1. Virus removal by membrane filtration

Table S6 shows virus concentrations before and after membrane filtrations. Filtrate samples were collected three times in one test, and the average concentrations were used to calculate LRVs. Only for UF-PES, concentrations of the first filtrate sample were used because the virus concentration decreased over time in the filtrate. Virus concentrations in UF filtrates are shown in Table S7. Table S8 shows all the virus LRVs by membranes calculated with equation 1.

Table S6. Virus concentrations in feed water/filtrate (log copies/ml)

| Membrane | Virus | 1st | 2nd | 3rd | 4th | 5th |
| --- | --- | --- | --- | --- | --- | --- |
| HL-0.1 | MS2 | 7.13/6.84 | 8.35/8.32 | 8.34/8.28 | - | - |
|  | PMMoV | 7.97/7.81 | 9.34/9.21 | 9.28/9.21 | - | - |
|  | AiV | 5.87/5.88 | 7.47/7.43 | 7.47/7.39 | - | - |
| HL-0.45 | MS2 | 6.73/6.53 | 8.18/8.42 | 8.17/8.38 | - | - |
|  | PMMoV | 7.85/7.71 | 9.21/9.39 | 9.04/9.39 | - | - |
|  | AiV | 5.80/5.77 | 7.33/7.41 | 7.32/7.45 | - | - |
| HB-0.45 (1L/min) | MS2 | 7.26/5.03 | 8.45/8.11 | 8.44/6.74 | 7.60/6.67 | 7.52/5.81 |
|  | PMMoV | 8.12/5.98 | 9.48/8.90 | 9.41/6.97 | 9.09/7.96 | 09.09/6.83 |
|  | AiV | 4.40/5.87 | 7.45/7.10 | 7.51/6.07 | 7.61/6.68 | 7.62/5.60 |
| HB-0.45  (2L/min) | MS2 | 7.57/5.88 | 7.39/7.31 | 7.20/6.57 | 8.71/7.88 | 8.67/7.31 |
|  | PMMoV | 9.30/7.23 | 8.97/8.77 | 9.11/7.57 | 9.73/9.22 | 9.77/8.28 |
|  | AiV | 7.52/5.99 | 7.56/7.40 | 7.65/6.34 | 6.94/6.11 | 6.89/5.51 |
| UF-1k | MS2 | 7.75/4.97 | 7.86/5.02 | 7.69/5.33 | 8.17/5.33 | - |
|  | PMMoV | 9.33/6.61 | 9.42/6.99 | 9.19/5.28 | 9.73/5.28 | - |
|  | AiV | 7.03/5.01 | 7.12/5.41 | 7.64/4.25 | 6.88/nd | - |

Table S7. Virus concentration in UF filtrates (log copies/ml)

| Run | Virus | 1^st^ sampling | 2^nd^ sampling | 3^rd^ sampling |
| --- | --- | --- | --- | --- |
| 1st | MS2 | 4.97 | nd | nd |
|  | PMMoV | 6.61 | 6.19 | 5.60 |
|  | AiV | 5.01 | 4.57 | 4.60 |
| 2nd | MS2 | 5.02 | 4.29 | nd |
|  | PMMoV | 6.99 | 6.30 | 5.62 |
|  | AiV | 5.41 | 4.76 | 4.18 |
| 3rd | MS2 | 4.43 | 3.85 | 3.68 |
|  | PMMoV | 5.60 | 4.96 | 5.02 |
|  | AiV | 4.25 | nd | nd |
| 4th | MS2 | 5.33 | 4.84 | 4.52 |
|  | PMMoV | 5.28 | 5.41 | 4.66 |
|  | AiV | nd | nd | nd |

Table S8. Virus LRVs by membranes

| Membrane | Virus | 1st | 2nd | 3rd | 4th | 5th |
| --- | --- | --- | --- | --- | --- | --- |
| HL-0.1 | MS2 | 0.29 | 0.03 | 0.06 | - | - |
|  | PMMoV | 0.17 | 0.13 | 0.08 | - | - |
|  | AiV | -0.02 | 0.03 | 0.08 | - | - |
| HL-0.45 | MS2 | 0.21 | -0.25 | -0.21 | - | - |
|  | PMMoV | 0.14 | -0.18 | -0.35 | - | - |
|  | AiV | 0.03 | -0.08 | -0.13 | - | - |
| HB-0.45 (1L/min) | MS2 | 2.22 | 0.33 | 1.70 | 0.93 | 1.71 |
|  | PMMoV | 2.14 | 0.57 | 2.44 | 1.13 | 2.26 |
|  | AiV | 1.58 | 0.36 | 1.44 | 0.93 | 2.03 |
| HB-0.45  (2L/min) | MS2 | 1.69 | 0.08 | 0.63 | 0.84 | 1.37 |
|  | PMMoV | 2.07 | 0.200 | 1.54 | 0.51 | 1.49 |
|  | AiV | 1.63 | 0.17 | 1.31 | 0.84 | 1.38 |
| UF-1k | MS2 | 2.77 | 2.84 | 2.36 | 2.84 | - |
|  | PMMoV | 2.72 | 2.43 | 3.91 | 4.46 | - |
|  | AiV | 2.02 | 1.71 | 3.39 | <1.56 | - |
